# Integrated Multiomics and Functional Validation Identify CLSPN as a Central Integrator of Genomic Instability and Immune Remodeling in Colorectal Cancer

**DOI:** 10.1155/humu/4686848

**Published:** 2026-10-26

**Authors:** Peng Wang, Jianyu Xia, Lexin Wang, Biao Hu, Haoran Jiang, Xinglan Wang, Xiaolu Fang, Xuekai Liu, Xiang Zhao, Xiaosong Li

**Affiliations:** ^1^ The Center for Clinical Molecular Medical Detection Innovative and Translational Laboratory of Molecular Diagnostics Laboratory Medicine Center The First Affiliated Hospital of Chongqing Medical University Chongqing China cqmu.edu.cn; ^2^ Key Laboratory of Clinical Laboratory Diagnostics (Ministry of Education) College of Laboratory Medicine Chongqing Medical University Chongqing China cqmu.edu.cn; ^3^ Department of Oncology Southwest Hospital Third Military Medical University (Army Medical University) Chongqing China tmmu.edu.cn; ^4^ Western Institute of Digital-Intelligent Medicine Chongqing China; ^5^ First Clinical College Affiliated First Hospital Chongqing Medical University Chongqing China cqmu.edu.cn

**Keywords:** CLSPN, colorectal cancer, genomic instability, replication stress, tumor immune microenvironment

## Abstract

Colorectal cancer (CRC) is characterized by pervasive genomic instability, yet the molecular mechanisms linking replication stress responses to genome alteration and phenotypic evolution remain incompletely understood. Here, we performed an integrative multiomics analysis combined with functional validation to investigate the role of CLSPN, a key adaptor of the ATR‐CHK1 pathway, in CRC. Machine learning–based feature selection identified CLSPN as a robust prognostic factor. Single‐cell and spatial transcriptomic analyses revealed that CLSPN is preferentially enriched in highly proliferative tumor cell populations and spatially localized within tumor core regions. Elevated CLSPN expression was strongly associated with increased copy number variation burden (FGA, FGG, and FGL) and chromosomal instability, indicating enhanced tolerance to replication stress and accumulation of genomic alterations. This genomic instability was further linked to remodeling of the tumor immune microenvironment. Functional assays demonstrated that CLSPN promotes proliferation, invasion, and migration while suppressing apoptosis in CRC cells. Collectively, these findings identify CLSPN as a key regulator of replication stress–driven genomic instability and highlight its role in shaping mutation‐associated tumor evolution, providing a potential target for therapeutic intervention.

## 1. Introduction

Colorectal cancer (CRC) is one of the most prevalent malignancies worldwide and remains a major cause of cancer‐related mortality [1]. Although advances in surgical techniques, systemic therapies, and molecular classification have improved patient management, outcomes for individuals with advanced disease remain unsatisfactory [2]. Notably, CRC exhibits substantial intertumoral and intratumoral heterogeneity, resulting in pronounced variability in disease progression, prognosis, and therapeutic responses [3]. Immunotherapy has recently transformed the therapeutic landscape for a subset of CRC patients, particularly those with mismatch repair‐deficient (dMMR) or microsatellite instability‐high (MSI‐H) tumors [4, 5]. However, the majority of CRC cases are microsatellite stable (MSS) and derive limited benefit from current immunotherapeutic approaches [6]. These observations indicate that conventional clinicopathological classification fails to fully reflect the biological complexity of CRC and highlight the need for more informative molecular determinants to better predict tumor behavior and therapeutic response.

Increasing evidence underscores the importance of the tumor microenvironment (TME) in CRC development and progression [7–10]. The dynamic interplay among immune cell infiltration, regulatory signaling networks, and stromal components not only facilitates tumor growth and dissemination but also critically influences treatment outcomes, particularly in the context of immunotherapy [11]. Advances in bulk, single‐cell, and spatial transcriptomic technologies have further revealed that tumor behavior is governed by a combination of intrinsic tumor programs, genomic instability, and the surrounding immune ecosystem [12, 13]. These insights emphasize the importance of investigating tumor biology through an integrated framework that considers both cell‐intrinsic mechanisms and microenvironmental interactions.

Claspin (CLSPN) is a key regulator of the DNA replication checkpoint and functions as an adaptor protein within the ATR‐CHK1 signaling pathway [14]. It plays an essential role in maintaining replication fork integrity, coordinating S‐phase checkpoint signaling, and orchestrating the DNA damage response, thereby safeguarding genomic stability under replication stress [15]. Given that replication stress is a defining feature of rapidly proliferating cancer cells, tumor dependence on ATR‐CHK1–mediated adaptive mechanisms has become a central theme in cancer biology [16]. Consistent with this, CLSPN has been reported to be aberrantly expressed in multiple cancer types and is associated with aggressive phenotypes, unfavorable prognosis, and resistance to therapy. Functionally, CLSPN supports tumor cell survival by enhancing tolerance to replication stress and facilitating cell cycle progression under genotoxic conditions [17–20]. Despite these findings, the role of CLSPN in CRC remains insufficiently characterized. In particular, whether CLSPN contributes to immune infiltration patterns, regulatory immune networks, spatial heterogeneity, and genomic instability—processes that collectively shape tumor evolution and treatment response—has not been comprehensively investigated [21–23].

In this study, we adopted an integrative multiomics approach combined with experimental validation to systematically investigate the role of CLSPN in CRC. By integrating transcriptomic, immune‐related, and single‐cell and spatial transcriptomic data, we examined its association with tumor immune contexture, spatial organization, and genomic instability. Furthermore, we evaluated its functional impact in vitro. Through these analyses, we aim to determine whether CLSPN acts as a central mediator linking replication stress adaptation, genomic instability, and TME remodeling, thereby establishing a unified mechanistic framework for CRC progression and therapeutic response.

## 2. Materials and Methods

### 2.1. Data Collection and Preprocessing

Transcriptomic and clinical data for CRC were obtained from TCGA‐COAD. Samples with incomplete clinical or survival information were excluded. Single‐cell data were retrieved from GEO (GSE108989), whereas CNV and somatic mutation data were obtained from TCGA. All datasets were curated and harmonized prior to analysis.

### 2.2. Construction and Evaluation of Prognostic Models

Prognostic models were developed using multiple machine learning approaches, including Lasso, Elastic Net, and Ridge regression. Model training was conducted with 10‐fold cross‐validation to determine the optimal regularization parameter (*λ*) and to reduce the risk of overfitting.

The prognostic performance of each model was evaluated using time‐dependent receiver operating characteristic (ROC) analysis, and the area under the curve (AUC) at 1‐, 3‐, and 5‐year time points was calculated using the timeROC package. The average AUC values across different models and time points were visualized using the ComplexHeatmap package. Based on the optimal prognostic model, a risk score was calculated for each patient, and individuals were stratified into high‐ and low‐risk groups according to the optimal cutoff value determined by the survminer package. Kaplan–Meier survival analysis and log‐rank tests were performed using the survival package and survfit function to compare survival differences between groups. Univariate and multivariate Cox proportional hazards regression analyses were conducted using the coxph function to evaluate the independent prognostic significance of the risk score. For multiple cohorts, we conducted a meta‐analysis using the inverse variance method and log hazard ratios (log HRs) as the main effect measures to comprehensively evaluate the prognostic value and robustness of the model in different datasets.

### 2.3. Feature Selection Analysis

To identify robust prognostic features, Lasso regression, Boruta, and random forest algorithms were applied for multidimensional feature selection. Lasso regression was implemented using the glmnet package, with the optimal *λ* determined by 10‐fold cross‐validation, and corresponding coefficient profiles and cross‐validation curves were obtained. The Boruta algorithm, based on an iterative random forest framework, evaluates feature relevance by comparing original variables with permuted shadow features, classifying them as confirmed, tentative, or rejected. Feature importance distributions and iteration results were visualized. Random forest analysis was conducted using the randomForest package, with model performance assessed by the out‐of‐bag (OOB) error. Variable importance was ranked according to the mean decrease Gini index. Features consistently identified across multiple methods were defined as candidate key genes.

### 2.4. Immune Infiltration and Immunoregulatory Analysis

To investigate the association between CLSPN expression and the tumor immune microenvironment, immune infiltration deconvolution analysis was performed using the CIBERSORT package to estimate the relative abundance of immune cell subpopulations in CRC samples. In addition, seven immune infiltration algorithms, including XCELL, TIMER, QUANTISEQ, MCPCOUNTER, EPIC, CIBERSORT‐ABS, and CIBERSORT, were applied to comprehensively evaluate the correlations between CLSPN expression and immune cell infiltration patterns. Spearman correlation analysis was conducted to assess the relationship between CLSPN expression levels and immune cell abundance, and the results were visualized using correlation heatmaps. Furthermore, samples were stratified into high‐ and low‐CLSPN expression groups to compare differences in immune cell infiltration between the two groups. For immune regulatory analysis, the expression profiles of immunostimulatory molecules, immunoinhibitory molecules, chemokines, and their corresponding receptors were systematically evaluated across different CLSPN expression subgroups. Integrated analyses of DNA methylation status and copy number amplification/deletion frequencies were further performed to comprehensively characterize the immune regulatory landscape associated with CLSPN expression.

### 2.5. Differential Expression and Functional Enrichment Analysis

To investigate CLSPN‐associated molecular features, samples were divided into high‐ and low‐expression groups based on predefined criteria. Differential expression analysis was conducted using the limma or DESeq2 package, with thresholds of |log2FC| > 1 and adjusted *p* < 0.05. Results were visualized using heatmaps. Survival differences between groups were evaluated by Kaplan–Meier analysis with log‐rank tests. Differences in CLSPN expression between tumor and normal tissues were assessed using the Wilcoxon rank‐sum test. To further explore the biological relevance of CLSPN, functional enrichment analysis was performed on DEGs identified between the two groups. Gene set enrichment analysis (GSEA) or other pathway‐based approaches were applied using Hallmark, KEGG, Reactome, or GO databases. Pathways with adjusted *p* < 0.05 were considered significant, with emphasis on cell cycle regulation, DNA damage response, and replication stress‐related pathways.

### 2.6. Single‐Cell RNA Sequencing Analysis

Single‐cell transcriptomic data were processed using the Seurat (v4.0) R package. Quality control was applied to the raw expression matrix to exclude low‐quality cells and those with excessive mitochondrial gene content. Data were then normalized, followed by dimensionality reduction, clustering, and cell type annotation. Clustering results were visualized using uniform manifold approximation and projection (UMAP), and cell identities were assigned based on canonical marker genes. The reliability of cell‐type annotation was further assessed by visualizing the expression patterns of representative canonical marker genes across the major cell populations. CLSPN expression across cell populations was visualized using FeaturePlot, VlnPlot, and DotPlot, allowing comparison of its distribution among different cell types. In addition, the expression patterns of representative genes, including TICRR, CENPF, and POLQ, were examined across clusters to further characterize CLSPN‐associated cell‐specific transcriptional features.

### 2.7. Spatial Transcriptomic Analysis

Publicly available spatial transcriptomic datasets generated using the 10× Genomics Visium Spatial Gene Expression platform were obtained from previously published studies. Spatial transcriptomic data were analyzed using the Seurat (v4.3.0) package in R. After quality control filtering, spots with low unique molecular identifier (UMI) counts, low detected gene numbers, or high mitochondrial gene proportions were excluded. Gene expression matrices were normalized using the SCTransform algorithm to minimize technical variability. Highly variable genes were identified using the FindVariableFeatures function, followed by principal component analysis (PCA) for dimensionality reduction. Shared nearest neighbor (SNN)–based clustering was subsequently performed using the FindNeighbors and FindClusters functions. UMAP was applied to visualize spatial transcriptomic heterogeneity. Spatial feature visualization and spatial distribution mapping were performed using the SpatialFeaturePlot function. Histopathological annotation was integrated with matched hematoxylin and eosin (H&E)–stained sections reviewed by experienced pathologists to distinguish tumor‐associated and nontumor regions. Tumor‐associated regions were defined based on the colocalization of malignant epithelial signatures, proliferation‐related transcriptional activity, and pathological tumor areas identified by histological evaluation, whereas the remaining regions were annotated as nontumor regions.

### 2.8. Cell Culture

Human CRC cell lines DLD‐1 (OriCell, H1‐1101) and HCT116 (OriCell, H1‐0401) were cultured in RPMI‐1640 and DMEM media, respectively, supplemented with 10% fetal bovine serum (FBS) and 1% penicillin–streptomycin. Cells were maintained at 37°C in a humidified incubator with 5% CO_2_.

### 2.9. Cell Transfection

Based on the baseline expression levels of CLSPN in different CRC cell lines, DLD‐1 cells with relatively high expression were selected for knockdown experiments, whereas HCT116 cells with lower expression were used for overexpression assays. Small interfering RNAs targeting CLSPN and the corresponding overexpression constructs were synthesized by MiaoLing Biotechnology (Wuhan, China). Cell transfection was performed using Lipofectamine 3000 (Vazyme, TL301‐01) according to the manufacturer′s instructions. Cells were harvested 24–48 h posttransfection for subsequent quantitative real‐time PCR (qRT‐PCR) validation and functional assays.

### 2.10. qRT‐PCR

Total RNA was extracted from cells using an RNA isolation kit (Vazyme, RC112‐01), and RNA purity and concentration were measured with a NanoDrop spectrophotometer. Complementary DNA (cDNA) was synthesized using a reverse transcription kit (Vazyme, R212‐01). qRT‐PCR was performed using a SYBR Green system, with *β*‐actin or GAPDH as internal controls. Relative gene expression levels were calculated using the 2^−*Δ* *Δ* *C* *t*^ method. Hu‐CLSPN‐F:TGAAAACTCAGGCAAGCAGCCTA; Hu‐CLSPN‐R:GGCTGATAGGATGGAATCGTGG.

### 2.11. Western Blot Analysis

Total cellular protein was extracted after CLSPN knockdown or overexpression, and protein concentrations were determined before electrophoresis. Equal amounts of protein were separated by SDS‐PAGE and transferred onto PVDF membranes. After blocking, the membranes were incubated with primary antibodies against CLSPN, E‐cadherin, N‐cadherin, vimentin, and GAPDH, followed by incubation with the corresponding secondary antibodies. Protein bands were visualized using an enhanced chemiluminescence detection system. GAPDH was used as the loading control.

### 2.12. Colony Formation Assay

Transfected cells were plated in sixwell plates at a density of 500–1000 cells per well and maintained for 10–14 days, with medium replaced every 2–3 days. After colonies became visible, cells were fixed with 4% paraformaldehyde and stained with crystal violet. Plates were then rinsed, air‐dried, and imaged, and colony numbers were quantified to evaluate proliferative and clonogenic capacity.

### 2.13. Transwell Invasion Assay

Cell invasive capacity was evaluated using Transwell chambers with 8 *μ*m pore size. Matrigel was diluted in serum‐free medium to a final concentration of 1 mg/mL, and 50 *μ*L of the diluted Matrigel was added to the upper chamber and incubated at 37°C for 30 min to allow solidification. Following transfection, cells were resuspended in serum‐free medium and seeded into the upper chamber, whereas the lower chamber was filled with medium containing 10% FBS as a chemoattractant. After 48 h of incubation, noninvading cells on the upper surface were removed. Cells that had migrated to the lower surface were fixed, stained, imaged under a microscope, and quantified.

### 2.14. Wound‐Healing Assay

Transfected cells were seeded in sixwell plates and cultured to approximately 90% confluence. A linear scratch was generated using a sterile pipette tip, followed by washing with PBS to remove detached cells. Serum‐free medium was then added, and wound closure was monitored at 0 and 48 h. The migration rate was quantified using ImageJ software.

### 2.15. Apoptosis Assay

Cell apoptosis was assessed using an Annexin V‐FITC/PI apoptosis detection kit (Biosharp, BL110A). Following transfection, cells were treated with apoptosis‐inducing agents (TNF − *α* + SM − 164) for 2–12 h. Cells were then collected after digestion with EDTA‐free trypsin, washed with PBS, and resuspended in binding buffer. Annexin V‐FITC and PI staining solutions were added according to the manufacturer′s protocol, followed by incubation for 15 min at room temperature in the dark. Apoptotic cells were quantified using flow cytometry, and data were analyzed with FlowJo software.

### 2.16. Statistical Analysis

Statistical analyses were performed using R software (Version 4.2.2) and GraphPad Prism (Version 8.0). Continuous data are presented as mean ± standard deviation (SD). Differences between two groups were evaluated using unpaired two‐tailed Student′s *t*‐test or the Wilcoxon rank‐sum test, as appropriate. Correlations were assessed using Spearman′s correlation analysis. Survival outcomes were analyzed using Kaplan–Meier survival curves and compared using the log‐rank test. All in vitro experiments were performed with at least three independent biological replicates. A two‐sided *p* value < 0.05 was considered statistically significant.

## 3. Results

### 3.1. Construction of a Robust Prognostic Model and Identification of CLSPN as a Key Gene in CRC

To establish a robust and clinically relevant prognostic model for CRC, multiple machine learning algorithms were systematically evaluated across independent datasets. Time‐dependent ROC analysis demonstrated variability in predictive performance among models; however, the selected model consistently achieved favorable accuracy at 1, 3, and 5 years. As shown in Figure 1A, it exhibited stable and high AUC values across cohorts, indicating strong predictive performance.

Figure 1Comprehensive evaluation of tumor prognostic models constructed by different algorithms. (A) Heatmap of average AUC values (1/3/5 years) across datasets (TCGA‐COAD‐OS), ranked by performance. (B) Coefficient heatmap showing gene weight and selection preferences (Lasso and Ridge). (C) Forest plot of meta‐analysis integrating HRs and 95% CIs across multiple studies. (D) Kaplan–Meier curves stratifying high‐ (red) and low‐risk (blue) groups (log‐rank test, *p* < 0.05).

**Figure:**
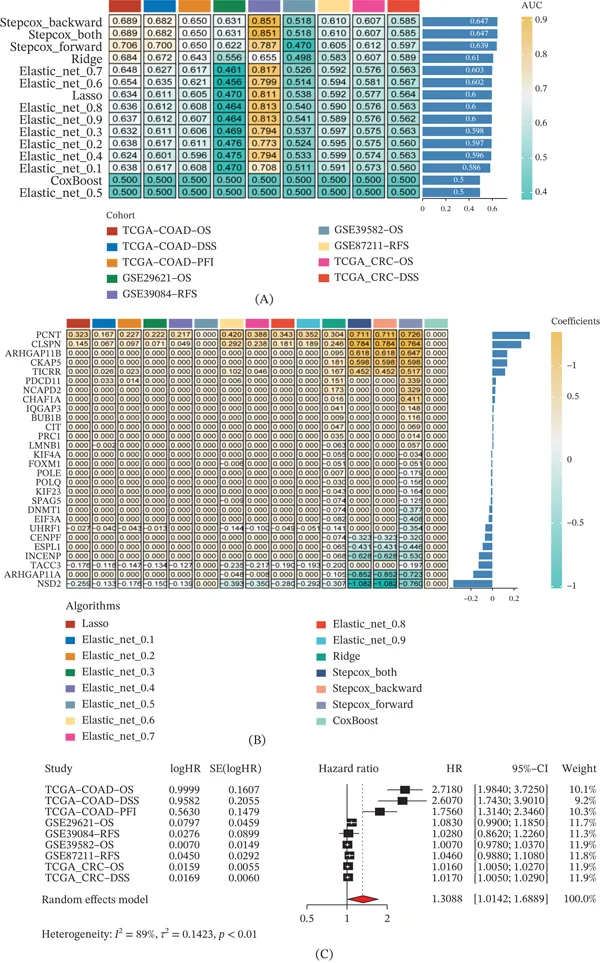

**Figure:**
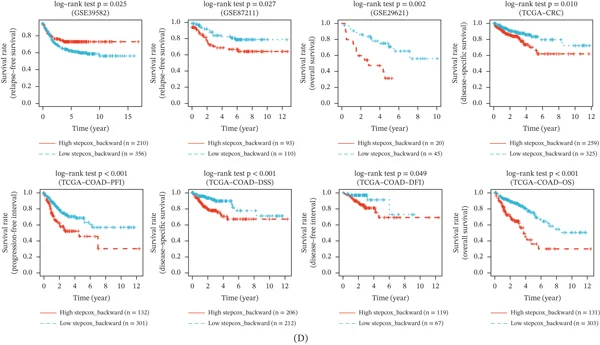

We next assessed gene contributions within the model. The coefficient heatmap revealed differences in feature selection across algorithms, whereas a subset of genes consistently retained nonzero coefficients (Figure 1B). Among these, CLSPN was stably selected and showed consistent weighting across models, highlighting its potential as a key prognostic gene.

Meta‐analysis across independent cohorts confirmed the prognostic significance of the model‐derived risk score. Patients in the high‐risk group exhibited significantly worse outcomes than those in the low‐risk group, with a pooled HR of 1.31 (95% CI: 1.01–1.69, *p* = 0.04) (Figure 1C). Kaplan–Meier analysis further demonstrated a markedly reduced overall survival rate in the high‐risk group (Figure 1D), supporting the strong prognostic stratification capability of the model.

### 3.2. Multialgorithm Feature Selection Identifies Robust Prognostic Candidates

To identify robust prognostic features, Lasso regression, Boruta, and random forest algorithms were applied for integrative feature selection. Lasso analysis with 10‐fold cross‐validation identified an optimal *λ* value of 13 corresponding to the minimal model error, with coefficients progressively shrinking as regularization increased (Figure 2A), indicating stable model convergence, effective feature selection, and reduced overfitting. Boruta analysis further evaluated variable importance through iterative comparison with shadow features, and genes categorized as “confirmed” were retained for subsequent analyses. CLSPN was identified as the most significant retained feature, with an importance score greater than 15 (Figure 2B), suggesting stable identification of prognosis‐related candidates. Random forest analysis showed that the OOB error reached a plateau with increasing tree numbers, indicating model convergence (Figure 2C). Feature importance ranking based on the mean decrease Gini metric further highlighted CLSPN as the top contributor, with an importance score exceeding 12. Collectively, these results establish a robust multialgorithm feature selection framework and consistently identify CLSPN as a core prognostic candidate gene for subsequent analyses.

**Figure 2:**
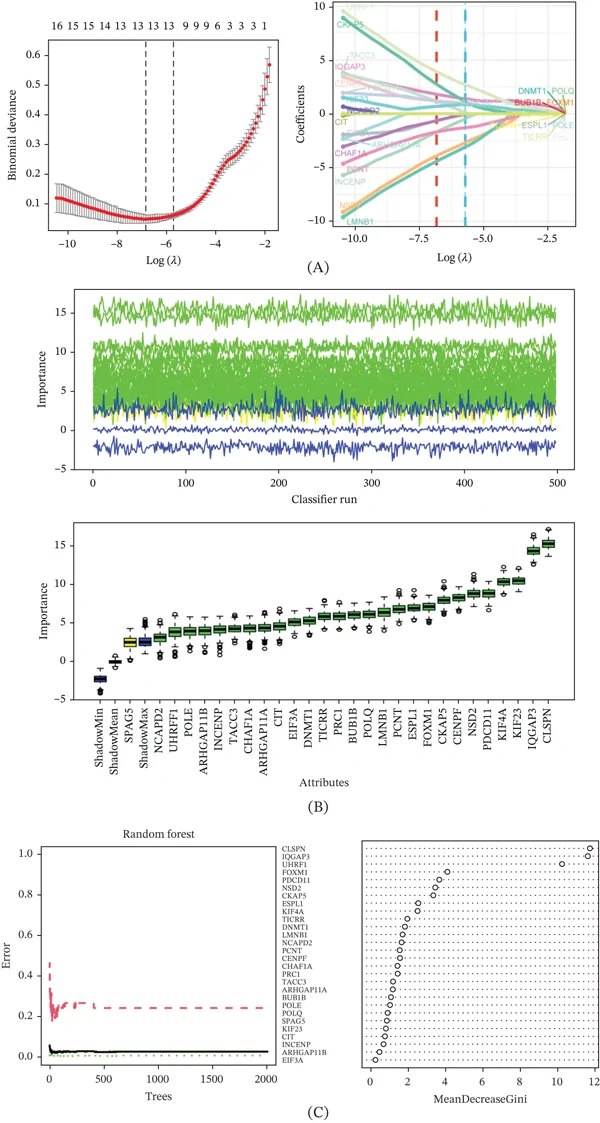
Systematic presentation of key parameter selection and feature screening. (A) Lasso regression cross‐validation error and coefficient path plots for optimal *λ* selection. (B) Boruta algorithm iteration plot and boxplot for feature importance and screening. (C) Random forest OOB error convergence and mean decrease Gini importance plot for key feature identification.

### 3.3. CLSPN Is Associated With Immune Infiltration and Immunoregulatory Networks in CRC

To investigate the immunological relevance of CLSPN in CRC, we first examined its association with immune cell infiltration. Spearman correlation analysis revealed that CLSPN expression was significantly correlated with multiple immune cell subsets, although the direction and magnitude of these associations varied across different algorithms (Figure 3A), indicating a complex relationship with the tumor immune microenvironment. We next assessed the association between CLSPN and immunoregulatory factors. Stratification by CLSPN expression quartiles demonstrated marked differences in immunoregulatory molecules at multiple levels, including gene expression, DNA methylation, and copy number variation (CNV) (Figure 3B), suggesting involvement in multilayered regulatory networks. Consistently, significant differences in immune cell infiltration were observed between CLSPN high‐ and low‐expression groups (Figure 3C), indicating that CLSPN expression is closely linked to immune infiltration patterns. Collectively, these findings support a strong association between CLSPN and the immune landscape of CRC, highlighting its potential role in linking tumor‐intrinsic genomic features with immune microenvironment remodeling.

Figure 3Multidimensional analysis of CLSPN in colorectal cancer. (A) Spearman correlation heatmap between CLSPN expression and immune infiltrating cells. (B) Immunoregulator characteristics grouped by CLSPN expression quartiles (Q1–Q4). (C) Heatmap of immune cell abundance differences (Wilcoxon test) between CLSPN high/low expression groups.

**Figure:**
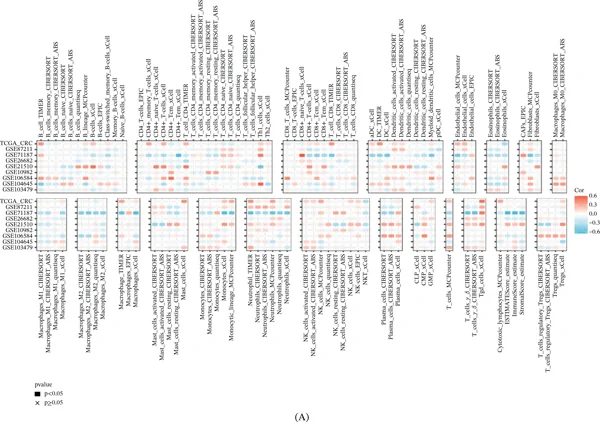

**Figure:**
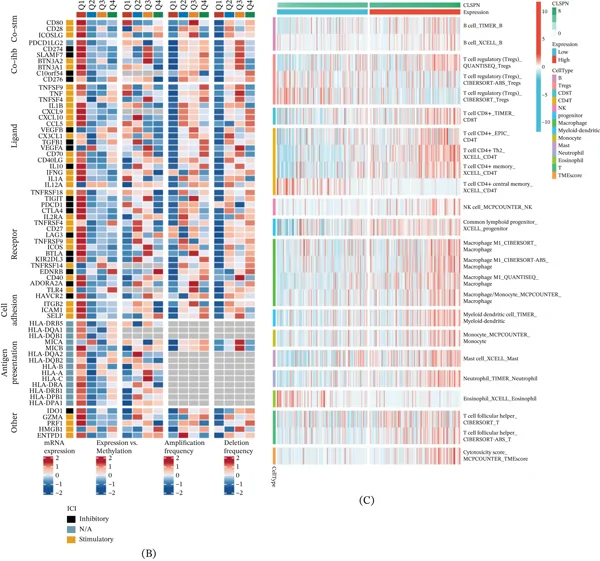

### 3.4. CLSPN Is Closely Associated With Clinical Phenotypes and Tumor‐Related Pathway Activation in CRC

Immune‐related gene expression differed between the CLSPN‐high and CLSPN‐low groups, particularly for immunostimulatory molecules, chemokines, immunoinhibitory molecules, and HLA‐related genes (Figure 4A). Analysis of the GSE106584 cohort showed significant differences in recurrence rates across CLSPN expression quartiles (*p* = 0.008), with the lowest recurrence proportion observed in the highest expression quartile (Figure 4B). CLSPN expression was significantly higher in tumor tissues than in normal tissues (Figure 4C). Pathway enrichment analysis revealed enrichment of DNA replication and repair‐related pathways, including homologous recombination, mismatch repair, and nucleotide excision repair, in the CLSPN high‐expression group (Figure 4D). Together, these results associate CLSPN expression with immune regulation, recurrence status, and DNA replication and repair processes in CRC.

**Figure 4:**
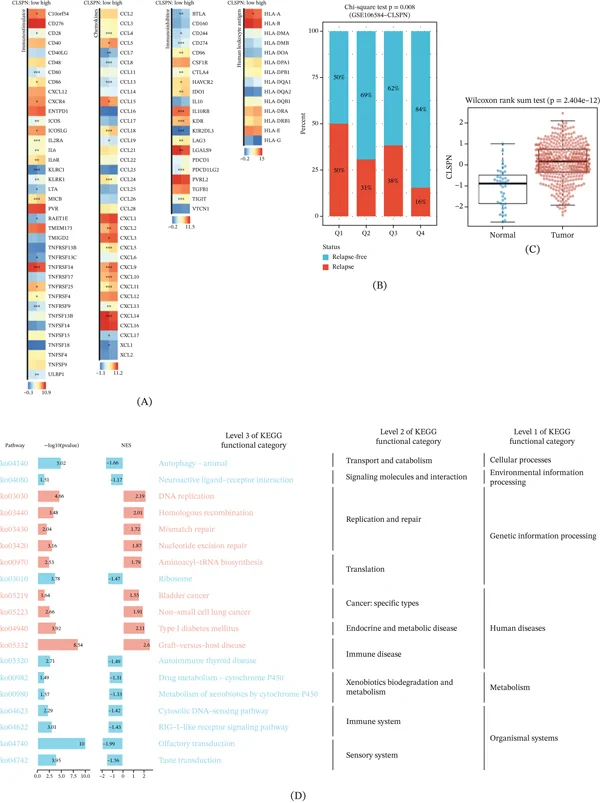
Clinical characteristics and pathway enrichment associated with CLSPN in colorectal cancer. (A) Heatmap of immune‐related gene expression in CLSPN‐high and CLSPN‐low groups. (B) Recurrence status across CLSPN expression quartiles (chi‐square test). (C) CLSPN expression in normal and tumor tissues (Wilcoxon test). (D) KEGG pathway enrichment and functional classification in CLSPN expression groups.

To delineate the cellular distribution of CLSPN in CRC, single‐cell transcriptomic data were analyzed. UMAP‐based clustering identified diverse cell populations within the TME, including CD4 Tconv, CD8 T, CD8 Tex, Tprolif, and Treg cells, reflecting pronounced cellular heterogeneity (Figure S1A). The expression patterns of representative canonical marker genes across the major cell populations were further visualized, supporting the reliability of the cell‐type annotations (Figure S1E).

CLSPN expression showed marked variability across cell clusters, indicating strong cell‐type specificity (Figure S1B). Notably, CLSPN was preferentially enriched in proliferation‐associated and tumor‐related cell populations, while remaining low in most nonproliferative immune subsets (Figure S1C), consistent with a proliferative cellular phenotype. Consistent patterns were observed for cell cycle–related genes, including TICRR, CENPF, and POLQ, which displayed similar enrichment across proliferative clusters (Figure S1D). This concordance further supports the association of CLSPN with actively cycling tumor cell populations. Overall, these findings demonstrate that CLSPN exhibits cell‐type–restricted expression in CRC, predominantly localized to highly proliferative tumor‐associated cells.

To further characterize CLSPN‐associated functional states, pathway activities were compared between CLSPN‐positive and CLSPN‐negative cells. Distinct patterns of pathway activation and suppression were observed across different cell populations, with significant alterations in multiple biological pathways within specific clusters (Figure S2A), indicating cell context–dependent functional roles. Spatial transcriptomic analysis further revealed that CLSPN displayed heterogeneous distribution across tissue sections. High‐expression regions were predominantly localized within tumor‐associated areas (Figure S2B), demonstrating pronounced spatial enrichment. Together, these results indicate that CLSPN integrates cell‐intrinsic functional programs with spatial tumor architecture in CRC.

### 3.5. CLSPN Expression Is Closely Associated With Genomic Instability in CRC

To assess the association between CLSPN and genomic alterations, we first examined genome‐wide CNV profiles in the TCGA‐COAD cohort. GISTIC analysis revealed widespread chromosomal gains and losses, reflecting a typical landscape of genomic instability in CRC (Figure 5A). We next evaluated the mutation features of CLSPN. Missense mutations represented the predominant alteration type, whereas other variants occurred at lower frequencies. Single nucleotide polymorphisms were the major form of variation, with C > T transitions being the most frequent subtype (Figure 5B), indicating a distinct mutational profile of CLSPN in CRC. To further explore the relationship between CLSPN expression and genomic instability, samples were stratified according to expression levels. Compared with the low‐expression group, the CLSPN high‐expression group exhibited significantly increased fraction of genome altered (FGA), fraction of genome gained (FGG), and fraction of genome lost (FGL) (Figure 5C), suggesting a strong association with elevated CNV burden.

**Figure 5:**
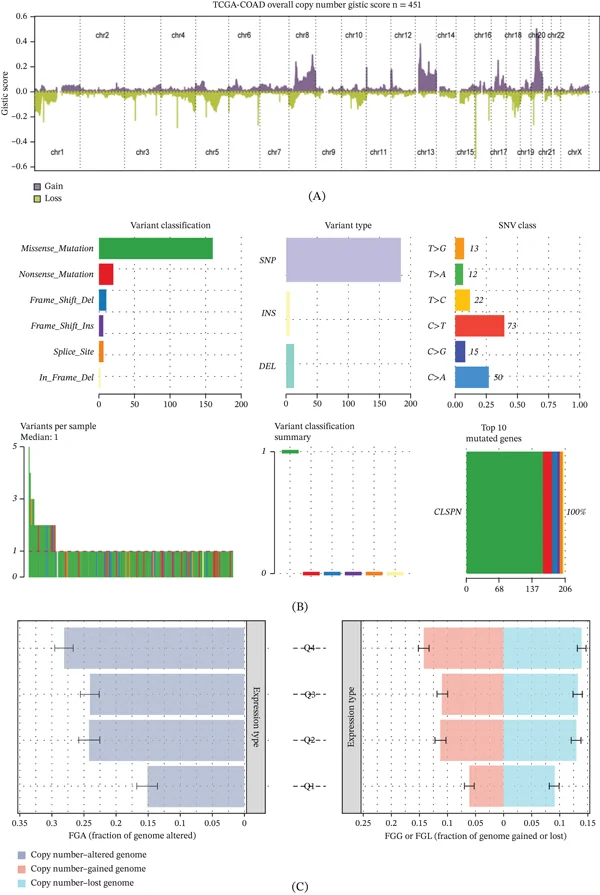
Genomic copy number, mutation types, and instability. (A) Genome‐wide CNV GISTIC map showing amplification (chr8/13/20) and deletion (chr1/5/10/18) patterns. (B) Summary of CLSPN mutation landscape (predominantly missense and C > T SNVs). (C) Association between CLSPN expression and genomic instability metrics (FGA, FGG, and FGL; ANOVA test).

### 3.6. Functional Experiments Verify That CLSPN Promotes Malignant Phenotypes in CRC Cells

To validate the functional role of CLSPN in CRC, its basal expression was first examined across CRC cell lines. CLSPN expression was relatively higher in DLD‐1 cells and lower in HCT116 cells, supporting the use of DLD‐1 for knockdown and HCT116 for overexpression experiments (Figure 6A). Efficient modulation of CLSPN expression was confirmed by qRT‐PCR (Figure 6B) and at the protein level by Western blotting (Figure 6F). Functional assays consistently demonstrated that CLSPN drives malignant phenotypes in CRC cells. Colony formation assays showed that CLSPN depletion markedly reduced clonogenic growth, whereas its overexpression enhanced proliferative capacity (Figure 6C). Wound‐healing assays revealed enhanced migratory ability upon CLSPN overexpression, whereas knockdown suppressed cell migration (Figure 6D). Transwell assays further indicated that CLSPN promotes invasive potential (Figure 6E). Western blot analysis further showed corresponding changes in the EMT‐related proteins E‐cadherin, N‐cadherin, and vimentin following CLSPN knockdown or overexpression, supporting an association between CLSPN and EMT‐related phenotypic changes (Figure 6F). In addition, flow cytometry analysis showed that CLSPN knockdown significantly increased apoptotic cell fractions, whereas its overexpression exerted an antiapoptotic effect (Figure 6G). These results provide direct functional evidence that CLSPN promotes proliferation, invasion, and migration while suppressing apoptosis in CRC cells, supporting its role as a driver of tumor progression.

Figure 6Functional effects of CLSPN on colorectal cancer (CRC) cell lines. (A) Baseline CLSPN mRNA expression levels in DLD‐1 and HCT116 cells, showing relatively higher expression in DLD‐1 cells and lower expression in HCT116 cells. (B) Validation of CLSPN knockdown in DLD‐1 cells and CLSPN overexpression in HCT116 cells at the mRNA level. (C) Colony formation assays evaluating the effects of CLSPN knockdown or overexpression on the proliferative capacity of CRC cells. (D) Wound‐healing assays evaluating the effects of CLSPN knockdown or overexpression on CRC cell migration. (E) Transwell invasion assays evaluating the effects of CLSPN knockdown or overexpression on CRC cell invasion. (F) Western blot analysis of epithelial‐mesenchymal transition (EMT)–related proteins following CLSPN knockdown or overexpression, including E‐cadherin, N‐cadherin, and vimentin. (G) Apoptosis assays evaluating the effects of CLSPN knockdown or overexpression on CRC cell apoptosis. Scale bars: 200 *μ*m in (D) and 50 *μ*m in (E).

**Figure:**
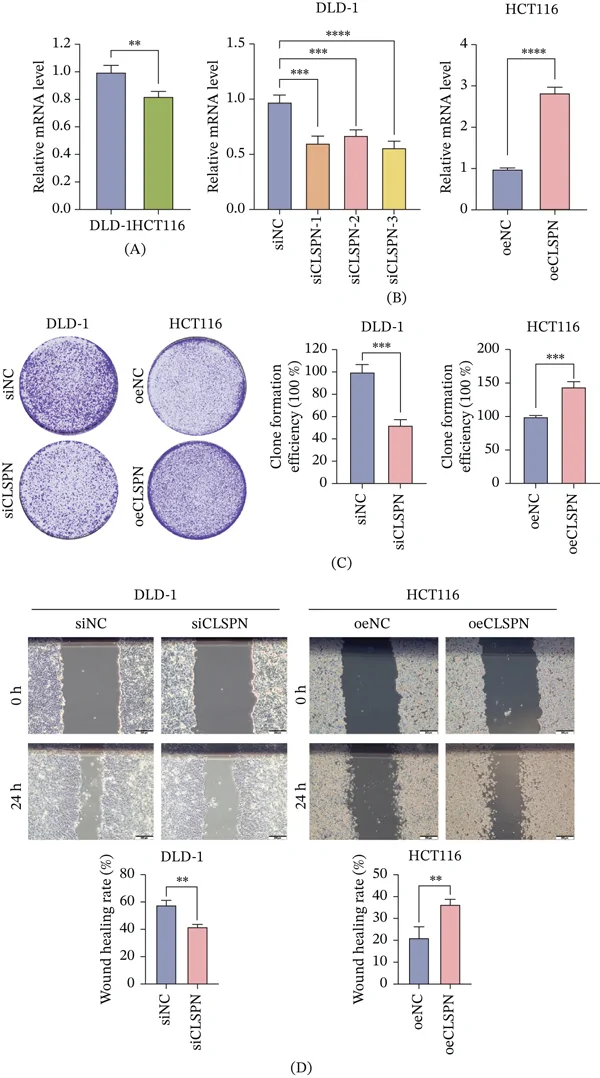

**Figure:**
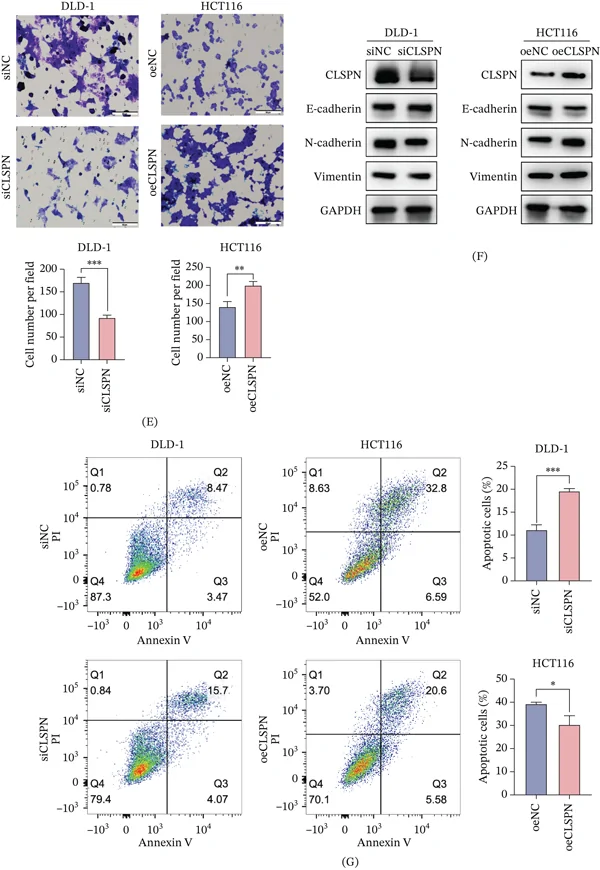

## 4. Discussion

Integrative multiomics analysis combined with machine learning and functional validation identifies CLSPN as a key determinant of CRC progression. Beyond its prognostic value, CLSPN emerges as a central integrator linking replication stress adaptation, genomic instability, and TME remodeling.

Single‐cell and spatial transcriptomic analyses resolve the ecological niche of CLSPN within the tumor ecosystem. CLSPN is selectively enriched in highly proliferative malignant subpopulations rather than uniformly expressed, consistent with its role in coordinating ATR‐CHK1 signaling and replication fork stability under S‐phase stress [24]. Structural and biochemical studies have demonstrated that CLSPN acts as a scaffold for CHK1 recruitment and activation, thereby sustaining checkpoint signaling during replication stress [25, 26]. Spatial confinement of CLSPN‐high cells to the tumor core suggests that CLSPN defines a replication‐competent and stress‐adapted cellular state. This distribution supports a model in which aggressive tumor clones are organized within a core‐dominant architecture, consistent with previous observations linking CLSPN overexpression to tumor progression and spheroid formation capacity [27].

The coupling between CLSPN expression and genomic instability extends this model. Replication stress is a major driver of chromosomal instability and copy number alterations during tumor evolution [28, 29]. Beyond its canonical checkpoint function, CLSPN contributes to replication fork protection and stabilization, thereby enabling cells to tolerate high levels of replication stress [30, 31]. Notably, CLSPN‐dependent and ‐independent CHK1 activation mechanisms have been described under diverse stress conditions, highlighting its central role in replication stress adaptation [32]. This tolerance permits the persistence of genomically unstable clones, consistent with the elevated CNV burden observed in CLSPN‐high tumors. Such a “tolerance‐over‐fidelity” strategy may facilitate tumor evolution while sustaining proliferative capacity.

Genomic instability further shapes tumor‐immune interactions. Accumulated genomic alterations influence antigenicity and immune signaling, thereby remodeling the TME [33, 34]. Emerging evidence indicates that CLSPN is associated with immune regulatory networks and immunosuppressive phenotypes, including macrophage polarization and immune checkpoint activation [35, 36]. These findings support a model in which CLSPN links intrinsic genomic instability with extrinsic immune dysregulation, thereby contributing to tumor immune evasion.

Functional evidence further supports this model, as disruption of CLSPN compromises malignant phenotypes, is accompanied by EMT‐related molecular changes, and enhances apoptotic susceptibility, highlighting a dependency of tumor cells on CLSPN‐mediated stress tolerance. From a therapeutic perspective, these findings position CLSPN as a potential vulnerability within the replication stress response network. Targeting CLSPN may impair the ability of tumor cells to cope with replication‐associated damage while simultaneously reshaping immune responsiveness, offering a rationale for combinatorial strategies involving DNA damage‐inducing agents and immunotherapy, particularly in MSS CRC [15, 37].

Several questions remain unresolved in the present study. Although our cell‐based experiments supported a role for CLSPN in CRC progression, its effects in vivo have yet to be examined. The relationship between CLSPN expression and the immune microenvironment was largely inferred from transcriptomic data, leaving open the question of whether CLSPN directly influences immune cell behavior or reflects broader changes in tumor cell states. Similarly, the higher genomic alteration burden observed in tumors with elevated CLSPN expression does not establish a causal link between CLSPN‐mediated replication stress adaptation and chromosomal instability. Addressing these questions through in vivo models and targeted mechanistic experiments would help clarify the contribution of CLSPN to CRC progression.

In conclusion, this study systematically characterized the role of CLSPN in CRC through integrative multiomics analyses and functional validation. Our findings identify CLSPN as a key replication stress adaptation factor that links genomic instability to immune remodeling and drives malignant progression in CRC. By connecting replication stress‐associated genome alterations with TME reprogramming, CLSPN provides new insights into the interplay between intrinsic tumor programs and extrinsic regulatory networks in CRC. Overall, these results highlight CLSPN as a promising prognostic biomarker and potential therapeutic target, and suggest that targeting CLSPN‐associated pathways may offer new opportunities for the treatment of refractory CRC.

## Author Contributions

Peng Wang: conceptualization, investigation, formal analysis, and writing—original draft. Jianyu Xia: formal analysis, interpretation of results, and writing—review and editing. Lexin Wang: formal analysis and visualization. Biao Hu, Haoran Jiang, Xinglan Wang, Xiaolu Fang, and Xuekai Liu: investigation and data curation. Xiang Zhao: supervision and writing—review and editing. Xiaosong Li: conceptualization, methodology, funding acquisition, resources, supervision, and writing—review and editing. Peng Wang and Jianyu Xia have contributed equally to this work.

## Funding

This study was supported by the graduate supervisor team project of the First Affiliated Hospital of Chongqing Medical University (CYYY‐DSTDXM‐202405).

## Disclosure

All authors have reviewed the final version of the manuscript and approved to submit to your journal. All authors have agreed to publish this manuscript.

## Ethics Statement

All methods were carried out in accordance with relevant guidelines and regulations.

## Conflicts of Interest

The authors declare no conflicts of interest.

## Supporting information


**Supporting Information** Additional supporting information can be found online in the Supporting Information section. Additional analyses of CLSPN expression and its molecular associations are provided in the supporting figures. Figure S1 presents the single‐cell distribution of CLSPN across malignant and immune cell populations, together with the expression patterns of related genes (TICRR, CENPF, and POLQ) and marker genes used for cell‐type annotation. Figure S2 further illustrates differences in pathway activity between CLSPN‐positive and CLSPN‐negative cells and the spatial heterogeneity of CLSPN expression within tumor tissues. These findings provide additional context for the cellular and spatial characteristics of CLSPN in colorectal cancer (Figures S1 and S2).

## Data Availability

The transcriptomic and clinical data for all patients were obtained from TCGA and GEO databases.
