# Supplementary material for: Integrated Multiomics and Functional Validation Identify CLSPN as a Central Integrator of Genomic Instability and Immune Remodeling in Colorectal Cancer
Source: Hum Mutat. 2026 Oct 26;2026:4686848. doi: 10.1155/humu/4686848 (PMC13698647; doi:10.1155/humu/4686848)
Supplement: Supplementary file 1 [file HUMU-2026-4686848-s001.docx]

**Supplementary figures**

Supplementary Figure 1. Expression characteristics and molecular associations of CLSPN at single-cell resolution. (A) UMAP clustering plot annotating major cell subpopulations (CD4 Tconv, CD8 T). (B) Expression density plot showing CLSPN enrichment in malignant proliferative cells. (C) Box/violin plots of CLSPN expression differences across cell subpopulations (Kruskal-Wallis test).(D) Violin plots comparing expression of core genes (TICRR, CENPF, POLQ) in immune and malignant cells. (E)

Heat map of annotated gene expression for major cell types such as CD4 Tconv and CD8 T cells.

**Supplementary Figure 2. CLSPN pathway regulation and spatial distribution patterns**. (A) Bubble plot of cell type-pathway activity differences (CLSPN-positive vs. negative). (B) Spatial transcriptomics heatmap displaying CLSPN expression heterogeneity in tumor tissue.
